# Tuberculosis treatment outcomes in Ethiopia from 2003 to 2016, and impact of HIV co-infection and prior drug exposure: A systematic review and meta-analysis

**DOI:** 10.1371/journal.pone.0194675

**Published:** 2018-03-19

**Authors:** Setegn Eshetie, Mucheye Gizachew, Animut Alebel, Dick van Soolingen

**Affiliations:** 1 Department of Medical Microbiology, School of Biomedical and Laboratory Sciences, College of Medicine and Health Sciences, University of Gondar, Gondar, Ethiopia; 2 Department of Nursing, College of Health Sciences, Debre Markos University, Debre Markos, Ethiopia; 3 National Institute for Public Health and the Environment (RIVM), Bilthoven, the Netherlands; Rutgers Biomedical and Health Sciences, UNITED STATES

## Abstract

**Background:**

Knowledge of tuberculosis (TB) treatment outcomes is substantially needed to assess the performance of national TB controls programs (NTPs). To date, the overall estimates of treatment outcomes have not been determined in Ethiopia. Therefore, this meta-analysis was undertaken to produce pooled estimates of TB treatment outcomes and to analyze the impact of prior anti-TB drug exposure and HIV co-infection.

**Methods:**

Potentially relevant studies were retrieved from PubMed, EMBASE, and MEDLINE online databases. The unpublished studies have been retrieved from the grey literature through Google and Google Scholar. The pooled estimates were calculated using random effect model. The summary estimates were also presented using Forest plots and Tables. The outcome measures were successful and unsuccessful treatment outcomes. Patients who were cured or with completed treatment defined as successful treatment outcome and patients meeting the definition of death, defaulting and failure are considered as unsuccessfully treated cases.

**Results:**

A total of 34 studies are included for meta-analysis. The pooled estimate of successful TB treatment outcomes amounts to 83.7% (95% CI 81.1%–86.3%). Of successfully treated cases, 33.9% were cured and the remaining completed cases. Besides, among patients with unsuccessful treatment outcome, nearly 50% were dead and the rest were treatment failures and defaulters. Sub-group analysis shows that high treatment success rate was estimated in Afar; 88.9% (95% CI 83.8%–94.2%), followed by Oromia; 88.5% (95% CI 82.6%–94.5%) and Gambella; 86.1% (95% CI 84.4%–87.9%), whereas relatively poor treatment outcome was noted in Tigray; 20.0% (95% CI 2.1%–37.9%) and Amhara; 19.0% (95% CI 12.6%–25.5%). The unsuccessful TB treatment outcome was found to be higher among HIV/TB co-infected cases with an odds ratio of 1.98 (95%CI, 1.56–2.52) and re-treated cases with an odds ratio of 2.17 (95%CI, 1.55–3.03). The time trend was assessed from 2003 to 2016, but it shows insignificant variation with treatment outcome (P = 0.108).

**Conclusion:**

The rate of successful treatment outcome in Ethiopia appears generally high, only slightly below the threshold suggested by the World Health Organization. History of tuberculosis treatment and HIV/TB co-infection were inversely associated with favorable treatment outcomes.

## Introduction

Since 1997,tuberculosis (TB) has been recognized as one of the major public health threats worldwide by the World Health Organization (WHO) and it replaced the ranks of HIV/AIDS as the leading causes of death from an infectious disease [1]. Though there are aggressive global strategies in place, TB control remains a challenge, particularly in resource-limited countries [2]. According to the last fourWHO reports, the global incidence of TB shows a steady decline [1,3–5]. However, in 2012 alone, an estimated 8.6 million new TB cases and 1.3 million deaths (23% of deaths reported among HIV positives) have been reported [3]. These figures increased to an estimated 10.4 million new TB cases (12% of cases were HIV positives) and 1.4 million deaths (29% of deaths reported represented HIV positive individuals) in a recently published global report [1].

It is noted that Ethiopia is one of the 22 countries with a high burden of TB, which together account for 80% of all global TB burden [1,6]. It is estimated that Ethiopia had 191,000 new TB cases in 2015. This number ranks Ethiopia 10^th^ globally and 4^th^ in Africa, after Nigeria, South Africa and the Democratic Republic of theCongo. Although there are remarkable achievements in the reduction of TB mortality from 94 to 33 per 100,000 population in the period from 2004 to 2014, TB still causes more than 30,00 fatalities per annum and more than 80 TB associated deaths every day [1,4]. Moreover, Ethiopia is also one of the 27 countries with a high burden of multidrug-resistant tuberculosis (MDR-TB). Based on a recent meta-analysis report, the pooled estimate of MDR-TB among new and previously treated cases was 2% (1 to 2%) and 15% (12 to 17%),respectively [7].

Ethiopia is the third largest and populous country in Africa. The Government of Ethiopia has nine ethnic-based administrative regions, which are referred to as the Regional States and two Federal City Administrations. Ethiopia has a population of about 101 million people, of which nearly 85% live in rural areas. It has huge topographic variation ranging from the lowest 116 meters below sea level to 4,620 meters above sea level. The WHO recommends the directly observed treatment, short-course (DOTS) strategy to scale up TB prevention and control [8]. Ethiopia has adopted this WHO-recommended DOTS strategy to improve TB treatment success rates and case detection rate [9]. However, pieces of evidence show that poor treatment adherence remains a major obstacle to fight TB epidemic in the country. The poor treatment adherence can be attributed to both organizational and personal related factors. It is largely known that low literacy levels, discriminatory behavior by health care professionals, self-denial due to stigma, long treatment duration and inaccessibility of public health facilities or shortage of drugs are some of the challenges compromising successful treatment outcomes [10,11].

The successful TB treatment coverage is one of ten priority indicators in achieving the milestones and targets of the End TB strategy. Globally, in 2000 TB treatment coverage was 36% (30–43%), but significant improvement has been reported in 2015; nearly 60% (50–70%). Among the 30 high TB burden countries, the highest levels of treatment coverage were noted in Brazil, China, Philippines, and Russian Federation. In the rest of the countries, including Ethiopia, TB treatment gaps remain especially among MDR-TB cases [12]. Global trends in TB treatment success rate shows a reduction from 87% in 2013 to 83% in 2014 [3,5]. The WHO report in 2015 claims that Ethiopia has achieved 90% treatment success rate, but the report indicates the validity of treatment outcome data remains in question and might not be the reflection of the reality [4].

Recent reports show a growing burden of HIV associated TB. This factor and also drug-resistant TB have been proposed as factors contributing to unsuccessful treatment [13,14]. Besides, limited evaluation of treatment outcomes in resource-limited countries like Ethiopia could comprise global TB control programs [3]. To date, the rates of TB treatment outcomes have been reported in several studies in Ethiopia. However, most of these surveys have presented local information. Comprehensive estimates of the extent of TB treatment outcome are needed for the programmatic management TB within the context of national TB control programs. Therefore, this meta-analysis was undertaken to produce pooled estimates of TB treatment outcomes in Ethiopia and to analyze the association of prior anti-TB drug exposure and HIV with these treatment outcomes.

## Methods

### Identification and selection of studies

Published and unpublished research reports describing TB treatment outcomes in Ethiopia were reviewed. Potentially relevant studies were identified through a literature search of PubMed, EMBASE, and MEDLINE online databases. All searches were conducted in January and February 2017 and all results were limited to English. Unpublished studies have been retrieved from the grey literature through Google and Google Scholar. The term ‘tuberculosis’ was searched with all of the following as a combination of free text and thesaurus terms in different variations: treatment outcome, treatment failure, successful treatment, unsuccessful treatment, mortality due to, HIV/AIDS and Ethiopia. The WHO documents were systematically searched on the respective website. The following keywords were used to retrieve studies from PubMed database; (Tuberculosis) AND (Treatment AND outcome OR (Successful AND Unsuccessful AND outcome)) AND (Associated AND Risk-factors OR (HIV AND Treatment-history)) AND (Ethiopia). The search was carried out by three authors (SE, AA, & MG), the most relevant studies were selected using predefined inclusion and exclusion criteria. The last author (DS) has checked the overall consistency of the searching process, study selection and exclusion.

Abstracts were reviewed from an initial search using defined inclusion and exclusion criteria. Any original study from Ethiopian settings was included in this review, whereas comments, editorials, and reviews were excluded. The articles were included if they estimated both successful and unsuccessful treatment outcomes in the total population of TB cases who started treatment, excluding those transferring out and/or those for whom outcomes were not reported. The systematic review and meta-analysis was carried out in accordance with Preferred Reporting Items for Systematic reviews and Meta-Analyses (PRISMA) guideline (**S1 Table**) [15].

### Outcome measures

First, the outcome measures of TB treatment were assessed as a percentage of successful and unsuccessful outcome among all patients who started anti-TB therapy, but transferring out patients were not included in the denominator. Successful outcomes included patients meeting the definition of ‘cure’ or ‘treatment completed’. Unsuccessful outcomes included patients meeting the definition of death, defaulting, and failure. Sub-group analysis was done by region (Amhara, Addis Ababa, Oromia, Southern region, Afar, Gambella and Tigray), HIV status, treatment category (new and retreated cases) and type of TB cases (pulmonary TB and Extra-Pulmonary TB). Second, the associations of HIV and prior anti-TB treatment with treatment outcomes were also measured. To describe time trends of successful treatment outcome in the country, we have used the index year as the year that the study was completed.

### Outcome definition

Treatment outcomes were defined according to the WHO criteria [3]. The successful treatment outcome is defined as patients who were cured or with completed treatment. Cured is a clinical and radiological improvement in a patient with a baseline smear positivity and evidence of at least two negative sputum smears, one during the maintenance period, and the other when the treatment was completed. Completed treatment is the completion of treatment during the predicted treatment period in patients with clinical and radiological improvements. Whereas unsuccessful treatment is defined as lost follow up (default), treatment failure, or death as an outcome measurement. Lost to follow up (default) are TB cases that did not receive medications for two months or more. Treatment failure is a patient that remains smear positive at month five or later during treatment. Death is an indicator of poor outcome and defined as a patient who died of TB or for any other reason during the course of treatment.

### Data extraction

Two authors (SE, AA) performed data abstraction using excel spreadsheet form. A third reviewer (DS) arbitrated any discrepancies between the two authors. From each study, the following parameters have been extracted: numbers of cured cases, treatment completed cases and successful treatments, and numbers of defaults, treatment failures, and deaths and unsuccessful treatments. Moreover, we have also extracted data on study location, region study year, study design, sample size, as well as, when available, potential covariates including the HIV status, treatment category (new and retreated cases), type of cases (pulmonary and extrapulmonary TB) were also summarized.

### Validity assessment

Studies were assessed for quality, with only high-quality studies included in the analysis. The quality of included studies has been assessed in accordance with Newcastle-Ottawa quality assessment scale [16]. Two authors (SE, DS) independently assessed the methodological quality, quality of reported data (extractable data to calculate treatment successful and unsuccessful rate), stratified data on the types of patient (pulmonary TB and extrapulmonary TB) and clear data research design of the included studies. After assessing the quality of each included study on the basis of these criteria, a composite quality score was assigned, ranging from 0 to 9. Studies scoring 6 and above were judged to be of high quality.

### Statistical analysis

A random effect meta-analysis was done to calculate the pooled estimates of TB treatment outcomes and 95% confidence intervals, using the approach of DerSimonian and Laird [17]. Freeman Tukey type arcsine square root transformation was used to address stabilizing variances [18]. The heterogeneity of studies was evaluated using the Q test and the I-squared statistic. To examine possible publication bias, Begg rank correlation and Egger weighted regression method has been employed; P<0.05 was considered indicative of statistically significant publication bias [19]. The effects of HIV/TB co-infection and prior anti-TB use were carried out and measured by odds ratio with 95% confidence intervals. To assess time trends of rates of treatment outcome, the rate of successful and unsuccessful treatment outcomes were plotted against the index year along with the observed percentages. Statistical analysis was done using Stata version 11 software package (Stata Corporation, College Station, TX). In all cases, P-values <0.05 were considered as statistically significant

## Results

**Fig 1:**
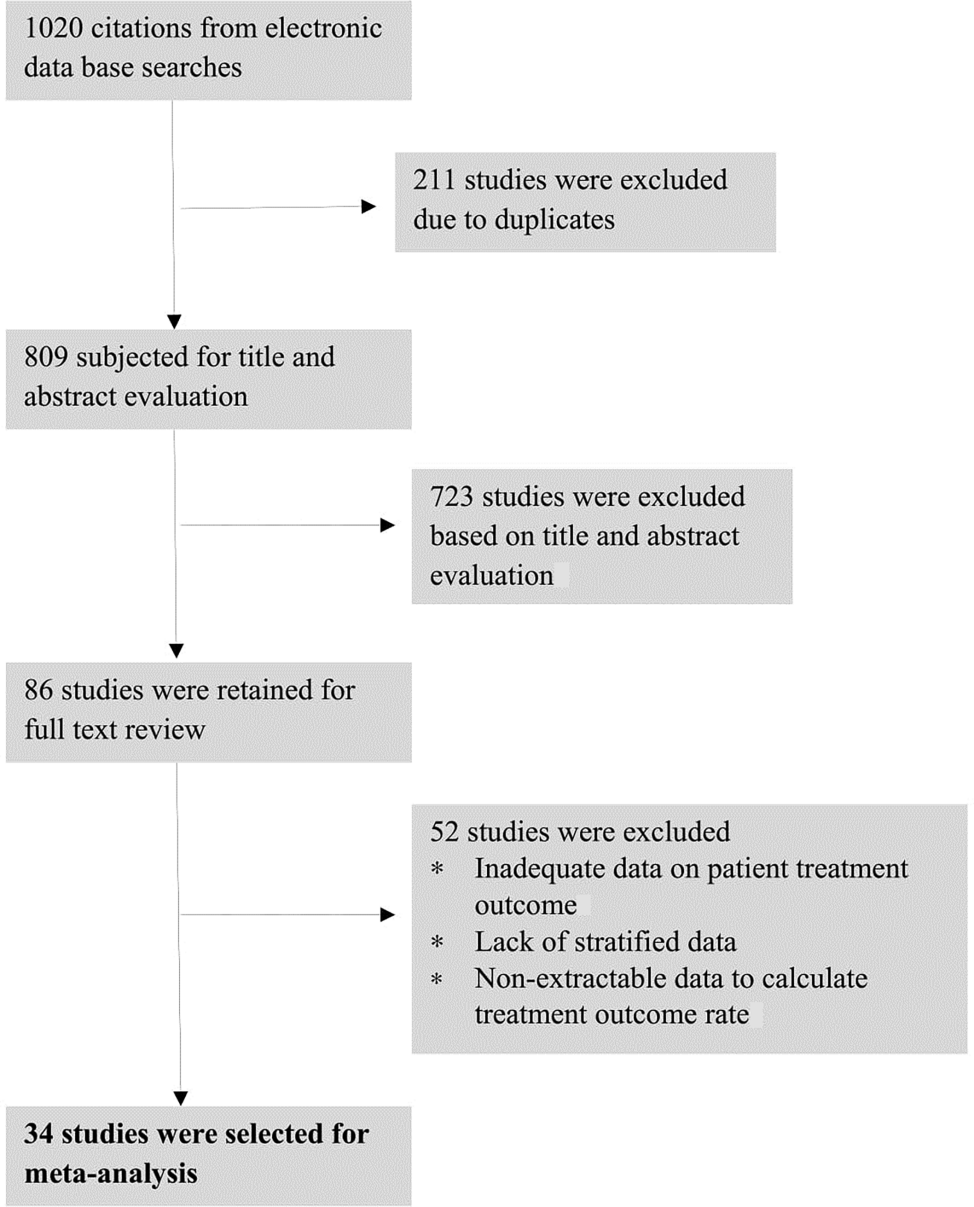
Flow chart indicating the result of literature search.

As indicated in **Fig 1** regarding the illustration of the search output, a total of 1,020 citations were identified. Of these, 812 were non-duplicate and subjected to further evaluation and 723 of them were excluded based on the title and abstract evaluation, 89 were retained on detailed full-text review. After full-text evaluation, 34 studies involving 60,138 TB patients from seven regions (Amhara, Addis Ababa, Oromia, Southern region, Afar, Gambella and Tigray) met the criteria for inclusion in the meta-analysis and are detailed in **S2 Table** [10,14,20–51]. Of the total population, 52,009 had successful treatment; the remaining 8,129 were unsuccessful treated cases. Nearly 50% of studies were conducted in Amhara region and the rest were done in other regions, but no finding was documented in Benishangul-Gumz, Somalia and Harar regions.

The included studies were published from the years 2009 to 2017 and 8 of the 34 studies were retrospective cohort studies; the rest concerned 26 retrospective cross-sectional studies. Of the eligible studies, 15 reported treatment outcomes in both new and retreated TB cases. Besides, 20 articles have also indicated data on the treatment outcomes of both HIV positive and negative patients.

### Treatment outcomes

**Fig 2:**
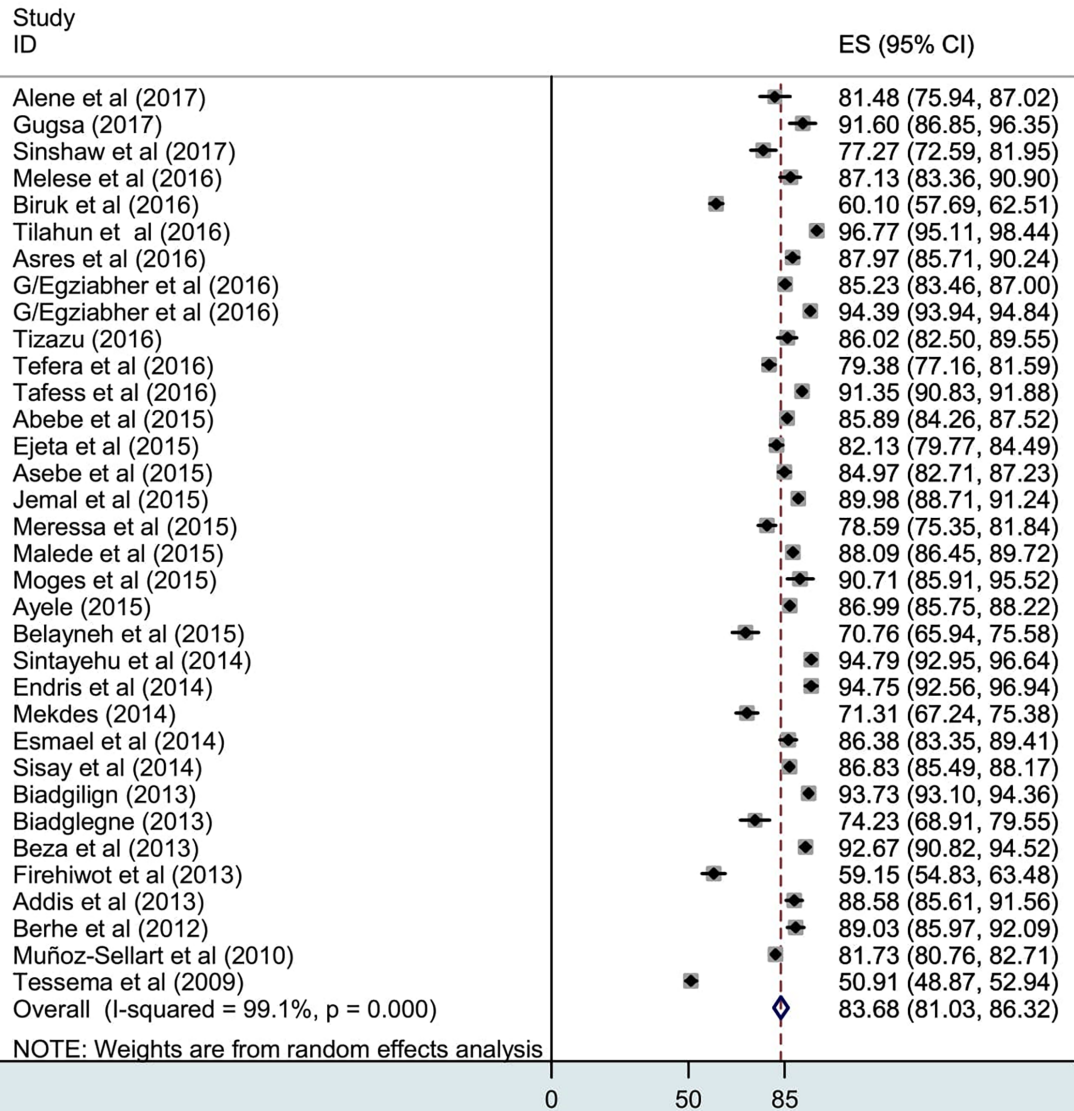
Pooled estimate of successful tuberculosis treatment outcome.

**Fig 3:**
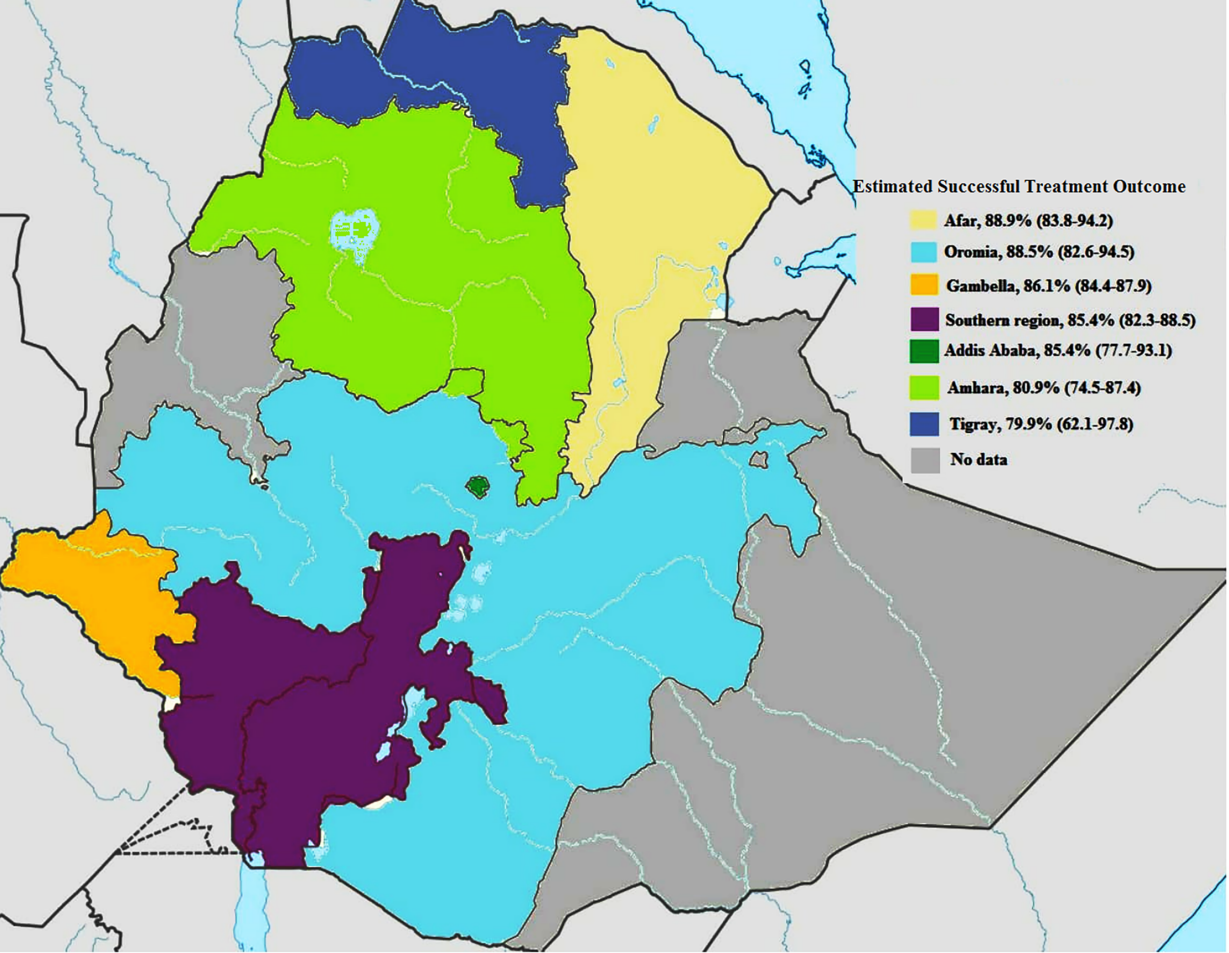
A sub-group analysis showing the estimate of successful treatment outcome in the regions of Ethiopia.

As detailed in a forest plot (**Fig 2**), the pooled estimate of successful TB treatment outcome was 83.7% (95%CI 81.1%–86.3%). High heterogeneity was observed, but Egger’s test yielded no indication of publication bias (I-squared, 99.1%). Based on sub-group analysis, highest treatment success rate was estimated from Afar (88.9%), followed by Oromia (88.5%) and Gambella (86.1%) (**Fig** 3). Of the successfully treated cases, 33.9% were cured cases, and 66.1% were treatment completed cases. Regionally, the relatively high cure rate was calculated in Addis Ababa (60.6%) followed by Tigray (56.4%) and Gambella (43.7%) (**Table 1)**. As shown in **Table 2**, success rate among new and retreated cases was 83% and 71%, respectively, and the difference is statistically significant (P<0.001). Likewise, treatment success rate was significantly lower among HIV positive (67%) cases compared to HIV negative cases (81%). Treatment success rates among patients with pulmonary TB and extra TB were also calculated, and the rate of successful treatment outcome is quite similar 78% in both cases.

**Table 1:** Tuberculosis treatment outcomes in the regions of Ethiopia.

Study region	Successful treatment		Unsuccessful treatment		
	Cured (%)	Treatment Completed (%)	Death (%)	Failure (%)	Default (%)
Amhara	25.4	74.6	55.9	4.9	41.5
Oromia	24.6	75.4	52.2	7.7	43.8
Southern region	27.9	72.0	34.5	2.3	63.1
Afar	42.0	57.4	28.7	18.3	50.5
Gambella	43.7	56.3	38.4	3.4	58.3
Addis Ababa	60.6	39.5	38.8	13.4	46.8
Tigray	56.4	43.6	62.6	18.8	17.4
**Overall estimate (95% CI)**	**33.9 (26.3, 41.5)**	**66.1 (58.5, 73.7)**	**48.9 (38.2, 59.7)**	**7.5 (5.5, 9.5)**	**45.1 (36.7, 53.5)**

**Table 2:** The proportion of tuberculosis treatment outcomes per patient characteristics.

Characteristics		Successful treatment (95%CI)	Unsuccessful treatment (95%CI)	P-value
HIV status	Positive	67 (56,79)	33 (21, 44)	0.04
	Negative	81 (75, 86)	19 (14, 25)	
Treatment category	New cases	83 (78, 88)	17 (12, 22)	<0.001
	Retreated cases	71 (65,78)	29 (22, 35)	
Patient category	Pulmonary TB	78 (73,82)	22 (18,27)	0.96
	Extra pulmonary TB	78 (72,84)	22 (16, 28)	

Of unsuccessfully treated cases nearly 50% of cases had death as the outcome, 45.1% were defaulters and the remaining 7.5% were patients with treatment failures. Briefly, 62.6% of unsuccessful treatment outcomes attributable to death were observed in Tigray, followed by Amhara (55.9%) and Oromia (55.2%). Relatively high treatment failure was also estimated in Tigray (18.8%) which is almost 8 times higher than an estimate noted in the Southern region (2.3%). Additionally, a high proportion of treatment defaulters has been calculated in the Southern region (63.1%); four times higher than the figure estimated in Tigray (17.4%) (**Table 1**). Besides, as indicated in **Table 2**; unsuccessful treatment rate among retreated cases was significantly higher than in newly diagnosed cases (29% versus 17%, P<0.001). Similarly, unsuccessful treatment rate among HIV positive and negative patients was 33% and 19%, respectively and the difference this also statistically significant (P = 0.04)

**Fig 4:**
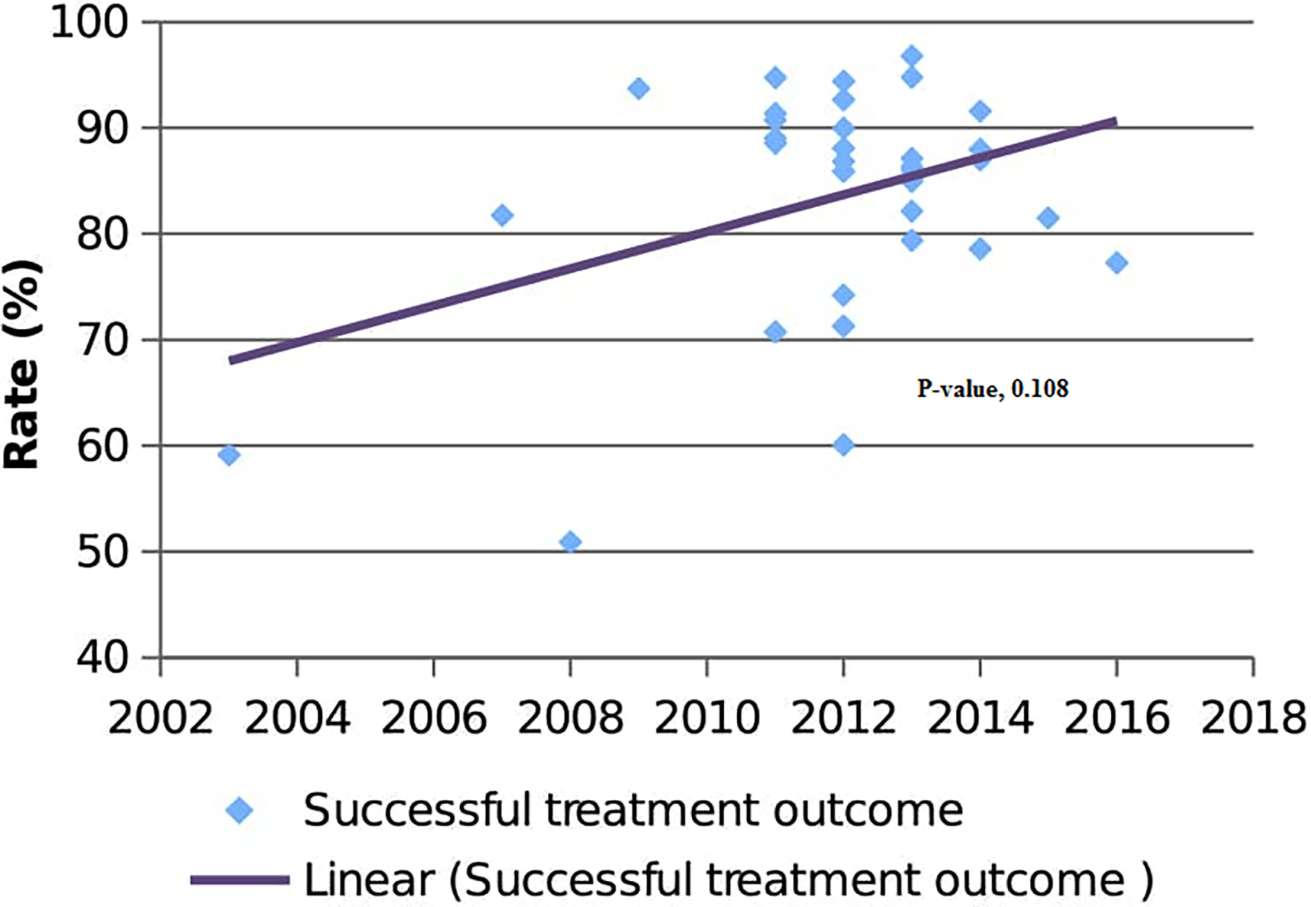
Time trend of successful TB treatment outcome.

Moreover, the time trend of successful treatment outcome was also assessed from the year 2003 to 2016 (**Fig 4**). There was a gradual increment in successful treatment outcome rate and equivalently there was also a decline time trend in unsuccessful treatment rate. However, no statistically significant time trend variation was observed (p = 0.108).

### Associations of HIV and prior drug exposure with TB treatment outcomes

**Fig 5:**
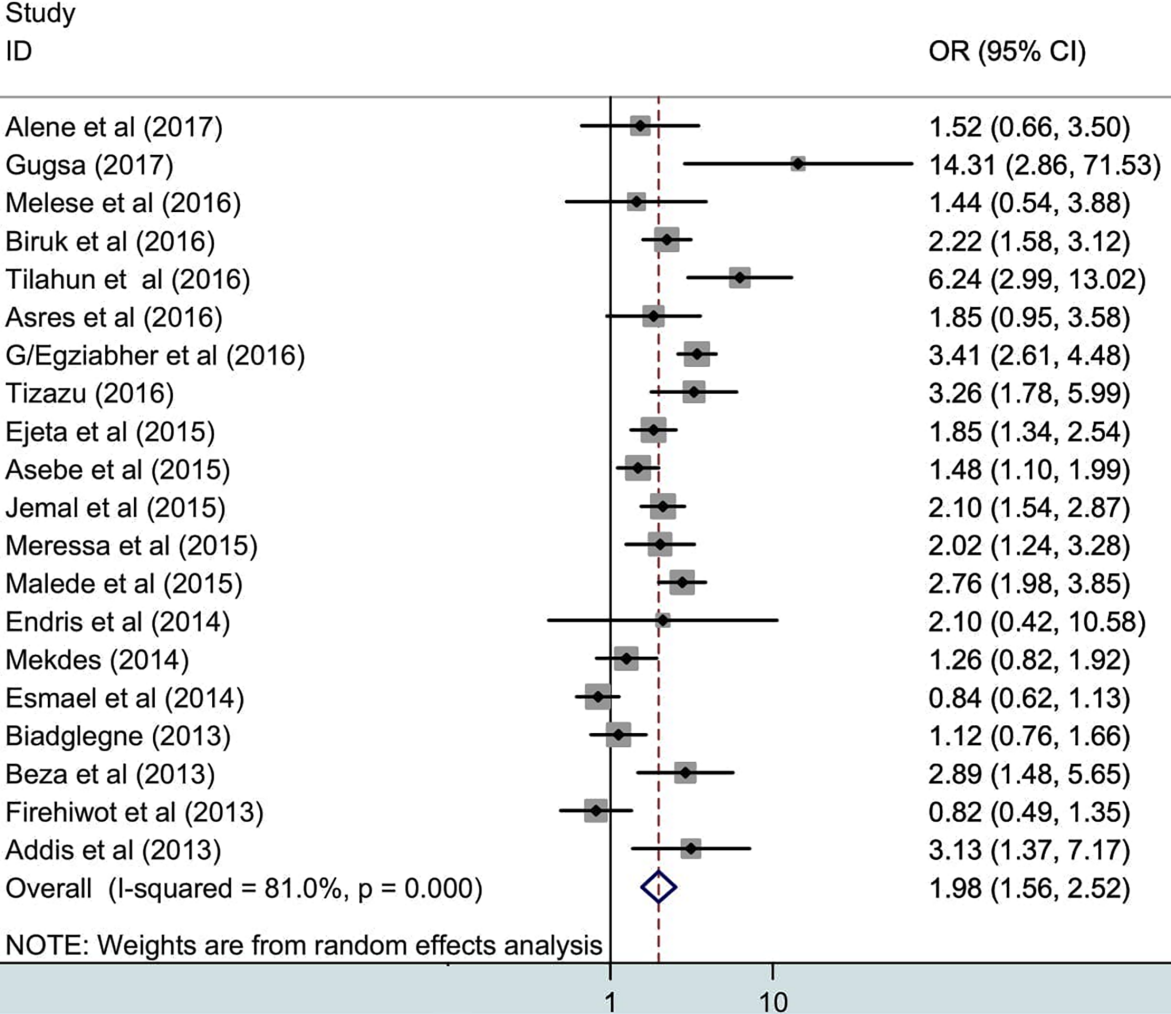
Pooled odds ratio indicating the association of HIV with poor treatment outcome.

**Fig 6:**
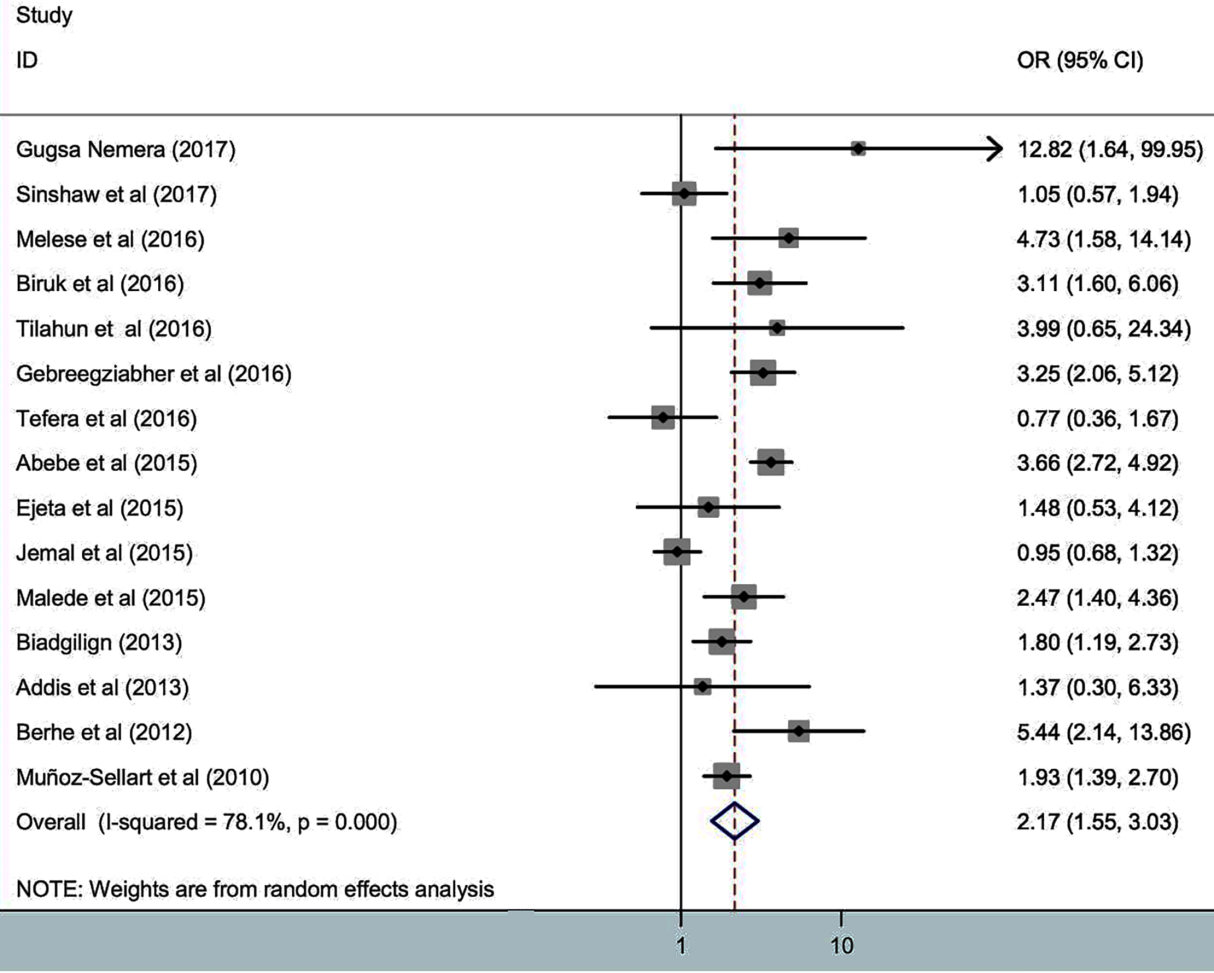
Pooled odds ratio indicating the association of prior anti-TB drug exposure with poor treatment outcome.

To further explore the associations of HIV and TB treatment outcomes, we used 20 studies which reported extractable data on the treatment outcomes of HIV positive and negative TB patients [20–22,25–28,30–36,39,41,43,48,49,51]. In **Fig 5** it is presented that unsuccessful treatment outcome was 1.98 times higher (95%CI, 1.56–2.52) in HIV/TB co-infected patients compared to HIV negative TB patients. Moreover, the association of poor treatment outcome with the previous history of TB treatment has been explored, a total of 15 studies have been used that have sufficient data to calculate the odds ratio [10,14,23,25–28,30,31,34,37,40,41,46,49]. Poor treatment occurrence was 2.17 fold higher (95%CI, 1.55–3.03) in retreated cases compared to newly diagnosed TB cases (**Fig 6**).

## Discussion

Diagnostic and therapeutic implementations based on the DOTS/Stop TB strategy have contributed to a significant success the control of TB [1,52]. However, TB treatment programs in most developing countries have not been effectively achieved [3]. Principally, poor treatment adherence is one of the setbacks in controlling of TB epidemic [53]. Non-adherence to treatment can lead to treatment failure, relapse, ongoing transmission and emergence of drug resistance strains [44]. Successful treatment requires coordination among the performance of national tuberculosis control programs (NTPs) and health care professionals in evaluating patient barriers to adherence and recommended DOT and the use of incentives and enablers that may assist the patient in completing the recommended therapy [1,47]. Cure rates in developing countries are frequently less than 50%; however, cure rates greater than 90% can be achieved when short-course chemotherapy regimens are given under strong supervision [9,54].

To date, TB treatment outcomes in Ethiopia have been assessed only in small and fragmented observational studies. Therefore, this is the first meta-analysis of its kind to summarize TB treatment outcomes reported in 34 studies. Hence, in the present analysis, the pooled estimate of successful treatment outcome was 83.68% (95% CI, 81.03% to 86.32%), and pooled unsuccessful treatment rate was 16.32% (95% CI, 13.67% to 18.97%). According to a recent WHO data in Ethiopia, however, the success rate was nearly 91% and the national wide report also claims the success rate was 91.4% [1]. A global report on antimicrobial resistance reveals that limited evaluations in resource-limited country notably Ethiopia could compromise the credibility of findings which leads to either overestimation or underestimation of results [55].

The results from this meta-analysis are comparable to treatment outcomes reported in South Africa (82.2%) [56], Iran (83.1%) [57], Kenya (82.4%) [58], and Ghana (87.7%) [59]. However, the treatment success rate obtained was lower than a report noted in Turkey (92.6%) [60]. This is as expected since high burdens of TB and MDR-TB are reported in Ethiopia. Besides, poor treatment adherence, inaccessibility of health care facilities and a shortage of rapid tests, could affect TB treatment outcomes. Surprisingly, lower treatment success rate was observed in Europe (74.4%) [61] compared to our findings. However, this study was done before 15 years, and therefore it is an old story and explored treatment outcomes before the introduction of Stop TB strategy, otherwise, it is largely speculated that TB control programs are increasingly implemented in the developed world, namely European countries. Moreover, previous meta-analysis studies have also determined treatment outcomes among MDR-TB patients; pooled treatment success rate was ranged from 43.7% to 65% [13,62,63]. Though a significant success rate is indicated in this study, previous reports have specifically meta-analyzed treatment outcome among patients with either MDR-TB or XDR-TB. Understandably, poor treatment outcome is largely implicated in patients with drug-resistant tuberculosis.

According to this meta-analysis of successfully treated cases, only one-third of the cases were cured which is extremely below the 85% threshold suggested by WHO. Relatively, the high cure rate was observed in Addis Ababa compared to other regions, was nearly 60%. In fact, accessibility of TB diagnostic tools, the presence of high-quality TB care services and significant skilled manpower are highly appreciated in Addis Ababa, have been known to increase TB cure rate. The NTPs is principally evaluated with the estimate of cured patients. Therefore, this finding draws attention to the urgent need for more effective control programs. Recently, WHO endorsed End TB strategy, which has superseded Stop TB strategy. The strategy aims to end the global TB epidemic with 90% reduction in TB deaths and 80% reduction in TB incidence by 2030 compared to the current scenario. Particularly, the strategy targets to address universal access to anti-TB drugs and to maximize TB treatment success rate more than 90% by 2025 [1,12]. It seems unachievable with these trends of experience, thus rigorous efforts are required from NTPs to accomplish targets that coincide with global strategy.

This study is also aimed to pinpoint TB treatment outcomes in the regions of Ethiopia. As it is noted, high treatment success rate was observed in Afar followed by Oromia and Gambella and least success rate was estimated in Tigray and Amhara. It is presumably due to limited research activities; only two studies were retrieved in regions with highest and lowest successful treatment outcome. Besides, the observed difference could be due to the fact that national tuberculosis and leprosy control programs are not equally implemented in the regions of Ethiopia, resulting in variation in the quality of notification and reporting of the treatment outcome data. Hence, high investment and equitable supervision of TB control services are substantially needed to harmonize the situation. Though Ethiopia has adopted the DOTS program since 2000 as an indispensable component for reducing TB epidemics, some regions (Benishangul-Gumuz, Harar and Somali) still have no data on TB control programs. It is suggested that TB control programs needed to have national-wide and representative data to accomplish global targets. Hence, assessment of TB treatment outcomes and monitoring and evaluation of the effectiveness of DOTS program are the necessity issues. Interestingly, future research projects need to be designed and expanded to figure out TB treatment outcomes with special emphasis to the above-mentioned regions.

Of the unsuccessful treatment outcomes, low defaults (17.4%) and high deaths (62.6%) were noted in Tigray region compared to others. Besides, the proportion of default cases was also high in South region (63.1%). As it is stated in the above, countrywide, TB care services are not uniform and the performance indicators of the treatment outcomes are not equally evaluated in the regions. Though there are significant improvements under the support of the DOTS program, it is largely relying on patient adherence and committed health professionals and possibly affects treatment outcomes. Moreover, it is also claimed that demographic factors, social factors, lifestyle factors and clinical factors might contribute high or low treatment outcome measures across the regions.

Several determinant factors have been identified that affect TB treatment outcomes [60,61]. The present study found that HIV/TB co-infection was associated with adverse TB treatment outcomes. As stated, the proportion of patients who had poor treatment was significantly higher among HIV positive cases than that among HIV negative individuals (33% versus 19%, P = 0.04). The occurrence of unsuccessful treatment outcome was 1.9 fold higher among HIV/TB co-infected cases compared to HIV negative TB patients. Ethiopia is one of high HIV/TB burden countries, so therapeutic challenges in TB control remain. Similar evidence has revealed that treatment outcomes of HIV/TB in co-infected patients differ from those patients who are infected with TB only [14]. Especially HIV/TB co-infected patients face various challenges, such as long duration of treatment, the frequency of drug administration, difficulty in management of drug interactions, adverse side effects of drugs and unregulated immunological responses so-called immune reconstitution inflammatory syndrome (IRIS). Reasonably, HIV/TB co-infected individuals are being treated for two infectious diseases and therefore the goals of treatment for both must be harmonized through therapy integration, use of concurrent Antiretroviral Therapy, management of HIV-related clinical problems, controlling drug toxicity, and monitoring of IRIS [64,65]. These measurements could bring optimal treatment outcomes and prevention of drug resistance.

A history of tuberculosis treatment is well known as one of the main contributing factors in poor treatment outcome compounded with drug resistance infections [14,60]. Accordingly, the present study demonstrates unsuccessful treatment outcome was higher among retreated cases compared to new cases (29% versus 17%, P< 0.001). Interestingly, poor treatment outcomes were 2.17 times more likely to occur among retreated TB cases compared to new cases. Likewise, a study claimed that history of defaulting was a contributing factor for re-defaulting. Furthermore, MDR-TB is commonly observed among previously treated TB patients. A recent meta-analysis study revealed that prevalence of MDR-TB in Ethiopia was 15% in retreated cases and 2% in new cases [7]. Several studies demonstrated that unsuccessful treatment is the main indicator of drug-resistant infections, notably MDR-TB. Therefore, this study underscores special emphasis needed to be given to re-treated TB cases to maximize favorable treatment outcomes. A retreatment strategy based on drug susceptibility testing and replacing the category II regimen may improve clinical outcomes.

Lastly, the present study has also assessed the time trend of TB treatment outcomes for the past nine years in Ethiopia. Stable time trend in both successful and unsuccessful treatment outcomes was observed. This was not expected since WHO declares Ethiopia has achieved the requirement of Millennium Development Goals in reducing TB incidence [1]. It has been noted that an increasing trend in optimal treatment outcomes is the main indicator of quality of TB control programs. However, based on the current study findings; there are long ways that NTPs needed to move forward to strengthen TB control programs. Recently, Stop TB strategy has replaced by End TB strategy that has incorporated more indicators that will be used to monitor progress. The strategy anticipated addressing more than 90% TB treatment coverage and success rate in worldwide basis by 2025 [12].

## Limitation of the study

One of the limitations of this study was the treatment outcomes of transfer out patients has not been determined since the included studies had not defined the outcomes for the aforementioned cases. The findings of this study were also limited by lack of stratified treatment outcomes data with special emphasis on child TB and MDR-TB cases. Besides, the results of this study may not represent the actual picture of the complete country, since data has not been obtained in Harar, Somali, and Benishangul-Gumuz. Lastly, methodological variations among included studies could also compromise the result of the study.

## Conclusion

The findings of the study suggest that successful TB treatment outcome in Ethiopia was generally good, but still below the 85% threshold defined standards. The study also demonstrates the proportion of cured cases was far from global expectations. There were significant variations in treatment outcomes across the regions of the country. Tuberculosis/HIV co-infection and history of previous TB treatment were found to have the greatest negative impact on treatment outcomes. Therefore, the findings draw special attention to regions with low successful treatment outcome, HIV/TB co-infected cases and re-treated treated cases.

## Supporting information

S1 Table PRISMA 2009 checklist. (PDF)

S2 Table Characteristics of the included studies. (PDF)

## Abbreviations

CI: Confidence interval
DOTS: Directly Observed Treatment, Short-course
HIV: Human immuno-deficiency virus
MDR-TB: Multidrug-resistant Tuberculosis
NTPs: National Tuberculosis Control Programs
TB: Tuberculosis
WHO: World Health Organization
