# Supplementary material for: Tuberculosis treatment outcomes in Ethiopia from 2003 to 2016, and impact of HIV co-infection and prior drug exposure: A systematic review and meta-analysis
Source: PLoS One. 2018 Mar 19;13(3):e0194675. doi: 10.1371/journal.pone.0194675 (PMC5858841; doi:10.1371/journal.pone.0194675)
Supplement: S2 Table — (PDF) [file pone.0194675.s002.pdf]

Table: Characteristics of included studies

Study name	Region	Study area	Study period	Study design	No TB cases	Cured	Treatment completed	Successful treatment	Died	failure	Default	Unsuccessful Treatment
Alene et al	Amhara	Gondar	2010 to 2015	RC	189	131	23	154	31	4		35
Gugsa Nemera	Oromia	Gimbi	2013 to 2014	RCs	131	22	98	120	6	0	5	11
Sinshaw et al.	Amhara	Gondar	2010 to 2016	RCs	308	32	206	238	31	2	37	70
Melese et al	Amhara	Debre Tabor	2008 to 2013	RCs	303	67	197	264	19	12	8	39
Biruk et al	Amhara	Gondar	2008 to 2012	RCs	1584	217	735	952	281	13	338	632
Tilahun et al	Addis Ababa	Addis Ababa	2009 to 2013	RCs	434			420	9	2	3	14
Asres et al	Southern	Bench Maji, Kaffa and Sheka	2008 to 2014	RCs	790			695				95
Gebrezgabiher et al	Southern	Dilla	2008 to 2013	RCs	1537	181	1129	1310	52	4	171	227
Gebreegziabher et al	Amhara	West Gojjam	2007 to 2012	RC	10094			9528	377	35	154	566
Tizazu Zenebea	Afar	Afar	2011 to 2013	RCs	372	128	192	320	17	1	34	52
Tefera et al	Amhara	Debre Birhan	2009 to 2013	RCs	1280			1016				264
Tafess et al	Afar	Afar	2002 to 2011	RC	11046	4245	5781	10,091	272	330	353	955
Abebe et al.	Oromia	Jimma	2008 to 2012	RC	1751	369	1135	1504	42	36	169	247
Ejeta et al	Oromia	Wollega	2009 to 2013	RC	1013	170	662	832	95	2	84	181
Asebe et al.,	Gambella	Gambella	2011 to 2013	RCs	958	262	552	814	43	4	97	144
Jemal et al.	Amhara	Metema	2009 to 2012	RCs	2155	445	1494	1939	88	21	107	216
Meressa D, et al	Addis Ababa	Addis Ababa	2009 to 2014	RC	612	396	85	481	85	10	36	131
Malede et al	Amhara	Wollo	2010 to 2012	RCs	1511	258	1073	1331	123	12	45	180
Moges et al	Amhara	Gondar	2007 to 2011	RCs	140	36	91	127	1	3	9	13
Ayele and Neko	Southern region	Gedeo Zone	2009 to 2014	RCs	2843	1042	1431	2473	161	12	197	370
Belayneh et al	Tigray	Tigray	2009 to 2011	RCs	342	43	199	242	88	5	7	100
Sintayehu et al	Oromia	Mizan	2010 to 2013	RCs	557	181	347	528	25	0	4	29
Endris et al	Amhara	Gondar	2007 to 2011	RCs	400	77	302	379	14	2	5	21
Mekdes Bekele	Addis Ababa	Addis Ababa	2009 to 2012	RC	474	262	76	338	39	37	60	136
Esmael et al	Amhara	Debre Markos	2008 to 2013	RCs	492	76	349	425	63	4		67
Sisay et al	Gambella	Gambella	2003 to 2012	RCs	2460	1177	959	2136	151	12	161	324

Biadgilign	Addis Ababa	Addis Ababa	2004 to 2009	RC	5687	1167	4164	5331	2	26	328	356
Biadglegne	Amhara	Bahir Dar	2010 to 2012	RCs	260	33	160	193	44	4	19	67
Beza et al	Amhara	Gondar	2007 to 2012	RCs	764	128	580	708	27		29	56
Firehiwot Cheru et al.	Amhara	Gondar univers	2002 to 2003	RCs	497	55	239	294	74	5	124	203
Addis et al	Amhara	Gondar	2008 to 2011.	RCs	438	89	299	388	41	1	8	50
Berhe et al	Tigray	Tigray	2008 to 2011	RCs	401	339	18	357	16	15	13	44
Muñoz-Sellart et al	Southern region	Southern Region	2002 to 2007	RCs	5995			4900	404	24	667	1095
Tessema et al	Amhara	Gondar	2003 to 2008	RCs	2320			1181	403	6	730	1139

Key: RC, retrospective cohort study; RCs, retrospective cross-sectional studies
